# Identification of novel drug targets and small molecule discovery for MRSA infections

**DOI:** 10.3389/fbinf.2025.1562596

**Published:** 2025-04-15

**Authors:** Nandha Kumar Subramani, Subhashree Venugopal

**Affiliations:** School of Bio Science and Technology, Vellore Institute of Technology, Vellore, Tamil Nadu, India

**Keywords:** *Staphylococcus aureus*, MRSA, subtractive proteomic data analysis, drug target, flavonoids, molecular docking, molecular dynamic simulation, MMGBSA

## Abstract

**Introduction:**

The topmost deadliest microorganism, namely, methicillin-resistant *Staphylococcus aureus* (MRSA), causes dreadful infections like bacteremia, pneumonia, endocarditis, and systemic inflammations. The virulence factors associated with MRSA exhibit multidrug-resistant characteristics, complicating treatment choices. So, the primary objective of this study is to identify the MRSA virulence factors and inhibiting its activity utilizing bioinformatic approaches.

**Methods:**

The screening of novel therapeutic MRSA targets was conducted based on the predictions retrieved from non-homologous, physicochemical analysis, subcellular localization, druggability, and virulence factor examinations. Following that, flavonoid compounds were docked against specific MRSA targets using AutoDock Vina. Further, molecular dynamic simulations and binding free energy calculations were performed using simulation software.

**Results:**

After examining 2,640 virulence factors that presented in MRSA, the heme response regulator R (HssR) was found to be a novel protein that greatly controls the levels of heme in MRSA infections. Subsequently, the binding energy calculations for flavonoid compounds and HssR revealed that the catechin provided −7.9 kcal/mol, which surpassed the standard drug, namely, vancomycin (−5.9 kcal/mol). Further, the results were validated by evaluating molecular dynamic simulation parameters like RMSD, RMSF, ROG, SASA, and PCA. Through analyzing these parameters, catechin provided a more stable, compact nature and less solvent exposure with HssR than vancomycin. Moreover, the predicted binding free energy for HssR-catechin was found to be −23.0 kcal/mol, which was less compared to the HssR-vancomycin (−16.91 kcal/mol) complex. The results suggested that the catechin was able to modulate the activity of the HssR protein effectively.

**Conclusion:**

These potential findings revealed that heme response regulator R as a promising therapeutic target while the flavonoid compound catechin could act as alternative therapeutic inhibitor that target MRSA infections.

## 1 Introduction

A wide variety of antimicrobial pathogens cause millions of human fatalities worldwide. To be precise, in 2022, antimicrobial-resistant pathogens caused 1.27 million deaths and 4.95 million infections in people (Medugu et al., 2023). Specifically, the drug-resistant microorganism, namely, methicillin-resistant *Staphylococcus aureus* (MRSA), was listed as the most lethal drug-resistant pathogen, responsible for over 100,000 deaths (Yao et al., 2023). In addition to that, the World Health Organization listed MRSA as a top-priority bacterium that causes severe infections in humans. Despite this, the death rate from MRSA had increased slightly each year since 2017 (Hossein Hasanpour et al., 2023). Furthermore, numerous studies over the last 2 decades have reported different prevalence rates of MRSA in various sectors, such as healthcare settings, overcrowded areas, cattle forms, and so on. Among them, the prevalence of MRSA mostly occurs in hospital environments (Shoaib et al., 2023). Further, it affects all age groups and causes mild to superficial infections. Primarily, this infection spreads through skin-to-skin contact, especially in individuals with weakened immune systems, ultimately resulting in fatality (Ahmed et al., 2023).

*Staphylococcus aureus* is a Gram-positive commensal bacterium and is known as an opportunistic pathogen. These bacteria are known for their resistance to most commercial beta-lactone antibiotics, including methicillin and penicillin (Bellis et al., 2024). It poses a significant global threat in healthcare, community, and livestock settings due to its antibiotic resistance, which is making the treatment more complex (Crespo-Piazuelo and Lawlor, 2021). Primarily, it is associated with nosocomial infection, skin and soft tissue infections, pneumonia, bloodstream infections, staphylococcal scalded skin syndrome, osteomyelitis, endocarditis, and bacteremia (Hou et al., 2023). Additionally, it can cause food poisoning by producing various toxins, including enterotoxins and exfoliative toxins (Leung et al., 2018), and also cause toxic shock syndrome by releasing superantigens into the bloodstream (Bukowski et al., 2010). The foremost mechanisms of MRSA resistance to most antibiotics are biofilm formation, the presence of penicillin-binding proteins (PBPs), and the presence of virulence factors, which play a major role in preventing the bacteria from all antibiotics (Wang et al., 2020). These virulence factors, including adhesion, capsule, exoenzyme, exotoxins, and colonization, are primarily involved in bacterial attachment to the host body. Besides that, the bacteria have various binding proteins that regulate various signaling pathways via the extracellular matrix in the host, including clumping factors, collagen-binding proteins, fibronectin-binding proteins, and elastin-binding proteins (Parveen et al., 2020).

Identifying the target protein that regulates MRSA infections in the host is a complicated process. Henceforth, there is a need to identify novel proteins that preserve the activity of MRSA. The identification of potential and novel therapeutic targets will improve the treatment of the infection with specific drugs. There are many approaches available for investigating drug targets in microorganisms. One of the unique methods to identify potential targets is subtractive genome analysis, otherwise called subtractive proteomic data analysis (Alhamhoom et al., 2022). This investigation predominately utilizes bioinformatics approaches and strategies that are cost-effective and speed up the identification of potential targets. This subtractive proteomic data analysis approach is primarily used for identifying potential therapeutic targets that are present in various life-threatening organisms, such as *Pseudomonas aeruginosa* (Uddin and Jamil, 2018)*, Streptococcus pneumoniae* (Khan et al., 2022)*, Helicobacter pylori* (Ibrahim et al., 2020)*, Mycobacterium tuberculosis* (Hosen et al., 2014)*, Edwardsiella tarda,* and *Salmonella enterica* (Hossain et al., 2017)*, Acinetobacter baumannii* (Kaur et al., 2021).

As of now, there have been a few antibiotics reported to treat MRSA infections. Currently, vancomycin is reported as the sole antibiotic that treats severe MRSA infections, but it has several side effects on the human body, like nephrotoxicity, ototoxicity, thrombophlebitis, and red men syndrome, which complicates its usage in infected individuals (Wang et al., 2022a). Apart from that, MRSA has also developed resistance to vancomycin due to its extensive use in treating infections. Therefore, there is a necessity to discover alternatives that mimic the activity of antibiotics. The researchers highlighted that the compounds derived from plants, like alkaloids, flavonoids, terpenoids, tannins, and so on, provide better inhibitory activity against MRSA infections. Among these phytochemical compounds, flavonoids have a wide range of properties, which include antioxidant, anti-inflammatory, anticancer, antimicrobial, and neuroprotective effects with low toxicity (Billowria et al., 2024).

Considering this potential promising action possessed by natural compounds, this investigation was taken up to *in silico* screening of potential flavonoids from a variety of natural sources against selected proteins from the subtractive proteomic data method to identify the candidates for developing new antimicrobial agents. Hence, the main objective of this study is to conduct a whole proteome analysis of methicillin-resistant *S. aureus,* aiming to identify and characterize new therapeutic targets and potential inhibitors against disease-causing proteins using *in silico* approaches.

## 2 Materials and methods

The detailed methodology is divided into two phases: Phase I involves identifying therapeutic targets from methicillin-resistant *S. aureus;* Phase II covers molecular docking, molecular dynamic simulation, and binding free energy calculations.

### 2.1 Phase I

#### 2.1.1 Proteome retrieval of MRSA strain

The complete protein sequences of *S. aureus* MRSA252 were retrieved from the NCBI (National Center for Biotechnology Information) database (https://www.ncbi.nlm.nih.gov/).

#### 2.1.2 Identification of paralogous protein

After retrieval of the proteins, the CD-HIT tool (Cluster Database at High Identity with Tolerance) was used to eliminate duplicate proteins that are presented in the data (Huang et al., 2010). The entire proteome set was subjected to CD-HIT (https://www.bioinformatics.org/cd-hit/), which was utilized to remove paralogous or duplicate sequences with a threshold value of 80%.

#### 2.1.3 Non homologous analysis

Furthermore, non-paralogous proteins were submitted to NCBI BLASTp (Basic Local Alignment Search Tool) (https://blast.ncbi.nlm.nih.gov/Blast.cgi?PAGE=Proteins) against *Homo sapiens* proteins, with an expectation value (E-value) of 10^−3^ to identify non-homologous proteins and homologous proteins. Afterward, only the non-homologous proteins were used for downstream investigations.

#### 2.1.4 Physicochemical characterization

The Expasy ProtParam server (https://web.expasy.org/protparam/) was used to compute the theoretical physicochemical properties of each protein, including molecular weight, isoelectric point, aliphatic index, instability index, and GRAVY (the grand average of hydropathicity). Proteins with an instability index lower than 40 were selected for further analysis because this classifies the protein as stable, while an instability index greater than 40 indicates the protein is unstable (Waterhouse et al., 2018). Only the stable proteins were chosen for the forthcoming steps.

#### 2.1.5 Protein localization prediction

To find therapeutic drug target identification, the identified proteins were subjected to PSORTb version 3.0.3 (https://www.psort.org/psortb/). These tools classified proteins into distinct types based on their cellular location, with five key locations in microbes, including cytoplasmic, extracellular, outer membrane, cytoplasmic membrane, cell wall, and unknown (Yu et al., 2010). Only the proteins present in the cytoplasmic were chosen for further investigation due to their established role in bacteria’s survival, antibiotic resistance, virulence, and potential ability to act as biomarkers or therapeutic targets.

#### 2.1.6 Druggability analysis of proteins

Furthermore, cytoplasmic proteins were subjected to druggability analysis. The Drugbank database (https://go.drugbank.com/) and Therapeutic Target Database (https://idrblab.net/ttd/) were used for comparing the druggability efficiency of proteins. The evaluation was carried out with an E-value cutoff of 10^−4^ along with default parameters (Craig et al., 2024; Zhou et al., 2024). Only the proteins (hits) with identified drug targets were selected for downstream analysis, while the no-hit proteins were excluded.

#### 2.1.7 Virulence factors and essentiality analysis

Identification of virulence proteins is an important step in the process of targeted drug therapy. These proteins play a major role in destabilizing the activity of immune cells in the host body, potentially leading to widespread disease (De Jong et al., 2019). For the determination of virulence proteins, the Blastp was performed using two common databases: VFDB (http://www.mgc.ac.cn/VFs/main.htm) and VICMpred (https://webs.iiitd.edu.in/raghava/vicmpred/). These two databases contained information about virulence factors, cellular processes, information molecules, and metabolism molecules (Liu et al., 2022). The proteins were shortlisted based on the comparison of hit proteins retrieved from these databases. Only the proteins that hit the VFDBs and VICMpred were used for further examinations. The virulence proteins retrieved from VFDBs and VICMpred were further screened for their essentiality in MRSA bacteria survival. For this purpose, the Database of Essential Genes (DEG) (http://origin.tubic.org/deg/public/index.php), an open-source database, was used. This resource contains information on experimentally verified essential genes across bacteria, archaea, and eukaryotes, as well as gene annotations, sequence information, protein details, essentiality classifications, and the experimental conditions that showed genes were necessary for microorganisms to survive. In this study, this database was used for identifying essential genes that were present in *S. aureus subsp. aureus* MRSA252 by submitting the FASTA sequences of retrieved protein. Afterwards, these protein sequences were compared to identify high-similarity protein sequences using the BLASTp tool, with an expectation value of 10^−5^.

#### 2.1.8 Metabolic pathway and protein-protein interaction network analysis

After selection of proteins through various filters from different databases, the specific metabolic pathways and protein-protein interactions were predicted through webservers. For identification of metabolic pathways, the KEGG (Kyoto Encyclopedia of Genes and Genomes) (https://www.genome.jp/kegg/) server was used for providing the functional annotations, and the protein-protein network analysis was predicted through String DB (Search Tool for the Retrieval of Interacting Genes/Proteins) (https://string-db.org/) (Szklarczyk et al., 2023). After identification of metabolic pathways and protein network analysis, the 3D structures of the protein were examined in the RCSB PDB (protein data bank) database.

### 2.2 Phase II

#### 2.2.1 Protein modeling and validation

After prediction the protein through subtractive proteomic data analysis, the 3D structure of the protein was modeled using the SWISS-MODEL web server SWISS-MODEL (expasy.org) (Waterhouse et al., 2018) and the 3D structure was validated by using SAVESv6-1 (https://saves.mbi.ucla.edu/) and ProSA webservers (https://prosa.services.came.sbg.ac.at/prosa.php). The SAVESv6.1 web server contains 3D structure validation tools, including PROCHECK, ERRAT, verify 3D, PROVE, and WHATCHECK. Among these, PROCHECK was utilized for analyzing the Ramachandran plot, while the ProQ (Wallner and Elofsson, 2003) (https://proq.bioinfo.se/cgi-bin/ProQ/ProQ.cgi) and ProSA (Wiederstein and Sippl, 2007) (https://prosa.services.came.sbg.ac.at/prosa.php) web servers predicted protein qualities as well as statistical parameters.

#### 2.2.2 Retrieval of flavonoid compounds

The flavonoid structures were retrieved from the NuBBE (research for the development and sustainable use of the biodiversity) database (https://nubbe.iq.unesp.br/portal/nubbe-search.html). This database contained a wide variety of compounds and secondary metabolites from plants, insects, fungi, bacteria, and marine organisms. It’s a user-friendly database that allows users to filter compounds based on chemical information, location, and biological properties (Pilon et al., 2017). Further, it enables users to retrieve similar substances by sketching the chemical structure of unknown compounds.

#### 2.2.3 Preparation of proteins and small molecules for molecular docking

After modeling the 3D structure of the protein and retrieval of flavonoid compounds, the protein and compounds were prepared before molecular docking studies. For the preparation of protein, the AutoDock version 1.5.6 tool was used, which involves removing water molecules, adding polar hydrogens, assigning Kollman charges, and converting the protein in pdb format to pdbqt format. For the preparation of ligands, OpenBabel GUI version 2.4.1 software (O’Boyle et al., 2011) was used for converting the compounds from sdf format to pdbqt format with default parameters (Kumar Subramani and Venugopal, 2024).

#### 2.2.4 Virtual screening using AutoDock vina

After preparation of the receptor and the ligands for molecular docking, virtual screening was performed using AutoDock Vina (ADV) software (Trott and Olson, 2010). The protein and ligand were subjected to Autodock 4.2.1, generating grid maps for blind docking and the grid box dimensions were 126 × 126 × 126 Å, with a spacing of 0.456. and center grid box is X = 0.913, Y = −0.034, and Z = 5.282. ADV software extensively investigates the lowest binding energy between protein and ligand complexes by employing a gradient-based optimization algorithm (Sanchez, 2013). In this study, ADV was used for predicting the binding interaction with multiple ligands on a single protein. After retrieving virtual screening results, the complexes with the lowest binding energy and highest hydrogen bond interactions were selected for molecular dynamic simulation. Further, the complex’s binding energies and binding free energies were compared with a commercial antibiotic drug, vancomycin. PyMOL version 2.5.4 (Yuan et al., 2017) and Discovery Studio Visualizer version 21.1.020298 software were used for the visualization of protein and ligand complexes.

#### 2.2.5 Molecular dynamic simulation

In this molecular dynamic simulation (MDS) step, the behavior of the protein in the presence of water (Native-Protein), the selected complex (Protein-Ligand), and the protein-vancomycin (Protein-Drug) complex were evaluated through 100 ns MDS using GROningen MAchine for Chemical Simulations (GROMACS) version 2023.2 (Gajula et al., 2016). The CHARMM27 all-atom force field was used to generate the protein topology file, and the default water model (TIP 3-point) was used to solvate the system in a cubic box, which mimicked the biological environment conditions. Then, the counterions, such as Na^+^ or Cl^−^, were added to neutralize the whole system. After adding ions, the energy minimization step was performed up to 50,000 ns steps using the steepest descent minimization algorithm. Simultaneously, the system underwent isothermal-isochoric (NVT) and isobaric (NPT) equilibration. Thereafter, the actual MDS was performed for 100 ns (Munieswaran et al., 2025). Through MDS results, root mean square deviation (RMSD), root mean square fluctuation (RMSF), the radius of gyration (ROG), solvent accessible surface area (SASA), principal component analysis (PCA), hydrogen bond (H-bond), and the binding free energy using gmx_MMBPSA were calculated for complexes. The graphs were plotted and visualized using Xmgrace software (Bucher et al., 2021). All these MDS were executed on the Ubuntu 20.04 LTS platform.

## 3 Results

### 3.1 Phase I

**FIGURE 1:**
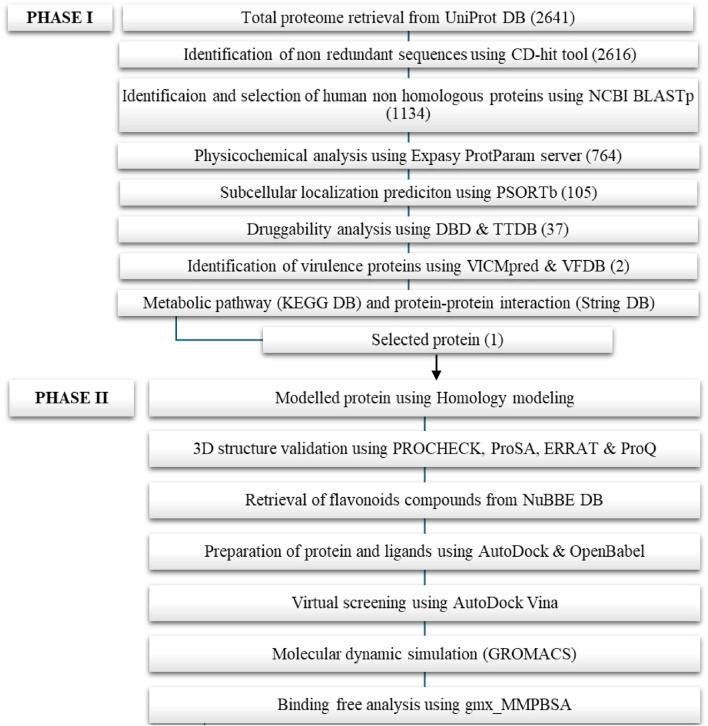
Schematic representation of Phase I and Phase II.

Identification of novel potential therapeutic targets against the methicillin-resistant *S. aureus* pathogen with subtractive proteomic data analysis was the main objective of the phase 1 study. The proposed drug targets were chosen based on druggability criteria, that includes non-homology to *H. sapiens* essential proteins for pathogens, contributes an important role in metabolic pathways, and act as virulence factors in proteins. Figure 1 shows the systematic workflow and their respective outcomes of the number of proteins from each step of phase 1 and phase II.

#### 3.1.1 Identification of non-paralogous, non-homologous proteins

The entire protein sequence of *S. aureus* (strain MRSA252) was downloaded from the UniProtKB database. A total of 2,640 proteins were retrieved, which include 888 reviewed proteins (Swiss-Prot), 1,753 unreviewed proteins (TrEMBL) and Taxon ID is 282458. Once the proteome was retrieved, the initial step was to remove paralogous proteins from the proteome. The paralogous proteins were removed due to their high sequence similarity, which potentially affects the outcome of the results. Following the elimination of paralogous proteins using the CD-HIT tool, only the non-paralogous proteins were retained for further analysis. The CD-HIT tool used 80% identity as the threshold value, which resulted in 2,616 non-homologous proteins. Next, human non-homologous proteins were identified by performing NCBI BlastP using 10^−3^ as the expected threshold value, and the maximum number of aligned sequences was found to be 5,000. Subsequently, each non-paralogous hit protein (2,616) was used against *H. sapiens*. The homologous proteins (1,482) and non-homologous proteins (1,134) were predicted based on the similarity between bacteria and humans. Further, the microbial proteins that were not similar to human proteins (non-homologous) were used for downstream analysis.

#### 3.1.2 Prediction of physicochemical characterization and subcellular localization

A total of 1,134 non-homologous proteins (Supplementary Sheet 1) were selected for physicochemical characterization. These proteins were submitted to the Expasy ProtParam web server to compute various physical and chemical parameters. This server calculates the instability index based on the categorization of stable and unstable classes. If the instability index exceeded 40, the server classified the protein as unstable; otherwise, this server classified it as stable (Chakma et al., 2023). Out of the 1,134 proteins, 764 proteins were classified as stable proteins (Supplementary Sheet 2). These stable proteins were chosen for subcellular localization analysis.

**FIGURE 2:**
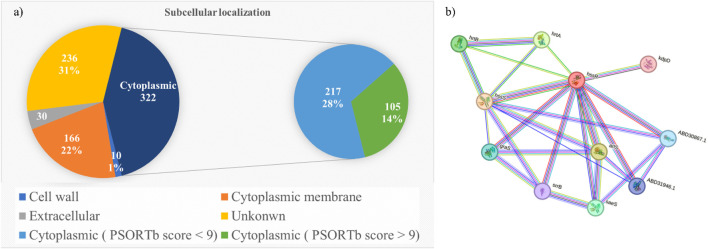
**(a)** Pie chart representation of subcellular localization of proteins. **(b)** Protein-protein interaction network of HssR protein.

For the subcellular localization prediction, 764 stable proteins were submitted to the PSORTp version 3.0.2 web server. Based on the localization score (Table 1), 322 proteins were identified in the cytoplasmic, 166 proteins in the cytoplasmic membrane, 10 proteins in the cell wall, and remaining 236 proteins were found to have an unknown localization (Supplementary Sheet 3). Among these, only the cytoplasmic protein with a prediction score greater than 9.0 was used for druggability analysis. Out of 322 cytoplasmic proteins with known localization, 105 (14%) proteins had prediction scores over 9.0. Potential vaccine targets could be extracellular proteins or membrane proteins, whereas cytoplasmic proteins are considered potential drug targets (Alhamhoom et al., 2022). The Figure 2A shows the pie chart illustration of subcellular localization of proteins. Further, these selected cytoplasmic proteins were used for druggability analysis.

**TABLE 1:** List of subcellular localization of selected MRSA proteins.

S. No	Subcellular localization	Number of proteins
1	Cell wall	10
2	Cytoplasmic membrane	166
3	Extracellular	30
4	Unknown	236
5	Cytoplasmic (PSORTb score <9)	217
6	Cytoplasmic (PSORTb score >9)	105

#### 3.1.3 Druggability and virulence factor analysis

Following the prediction of cytoplasmic proteins of MRSA, the druggability potential for each target was assessed. These shortlisted proteins were subjected to BLASTp against the DrugBank Database and Therapeutic Target Database with an expected threshold value of 10^−5^. By comparison of both databases, only the hit proteins were considered for drug-like properties analysis. Out of 105 proteins, only 37 were identified (Table 2) in both databases and were subsequently used for virulence factor analysis. The virulence factor database (VFDB) and the VICMpred web servers were utilized for identification of the virulence factors in selected protein sequences. From this analysis, two proteins were identified as virulence factors, which include penicillin-binding protein 4 (A0A7U7EUI7) and a heme response regulator (Q6GE73). Further, the proteins essentiality on MRSA survival were analyzed using BLASTp against the DEG database with an ID of DEG1065. By examining the results, both proteins were identified as non-homologous proteins, essential proteins that are involved in bacterial mechanisms, and drug-like proteins that are involved in MRSA regulations. Subsequently, the 3D crystal structures of these two proteins were examined in the Research Collaboratory for Structural Bioinformatics Protein Data Bank database (RCSB PDB). Among these two proteins, only the heme response regulator protein R was selected for Phase II investigation due to the absence of its 3D structure in the PDB.

**TABLE 2:** List of selected protein sequences through non homologous, cytoplasmic, essential druggability and virulence factor analysis. (DBD- DrugBank Database, TTDB- Therapeutic Target Database, VFDB- Virulence factor database).

S. No	UNIPROT ID	Seq. length	Instability index (ProtParam)	Subcellular localization		Druggability analysis		Virulence factor analysis	
				Psortb	Psortb score	DBD	TTDB	VICM pred	VFDB
1	Q6GK90	749	Stable	Cytoplasmic	10	YES	YES	Cellular process	NO
2	Q6GEJ6	61	Stable	Cytoplasmic	10	YES	YES	Cellular process	NO
3	Q6GG72	420	Stable	Cytoplasmic	10	YES	YES	Metabolism Molecule	NO
4	A0A7U7IBT3	235	Stable	Cytoplasmic	9.97	YES	YES	Cellular process	NO
5	Q6GEZ1	356	Stable	Cytoplasmic	9.97	YES	YES	Cellular process	NO
6	Q6GGU4	354	Stable	Cytoplasmic	9.97	YES	YES	Metabolism Molecule	NO
7	Q6GHF0	347	Stable	Cytoplasmic	9.97	YES	YES	Cellular process	NO
8	Q6GHQ2	449	Stable	Cytoplasmic	9.97	YES	YES	Cellular process	NO
9	Q6GHW1	160	Stable	Cytoplasmic	9.97	YES	YES	Metabolism Molecule	NO
10	Q6GI01	572	Stable	Cytoplasmic	9.97	YES	YES	Metabolism Molecule	NO
11	Q6GIL5	505	Stable	Cytoplasmic	9.97	YES	YES	Cellular process	NO
12	A0A7U7EV46	245	Stable	Cytoplasmic	9.97	YES	YES	Information and storage	NO
13	A0A7U7EVF1	549	Stable	Cytoplasmic	9.97	YES	YES	Cellular process	YES
14	A0A7U7EW44	589	Stable	Cytoplasmic	9.97	YES	YES	Virulence factors	NO
15	Q6GE73	224	Stable	Cytoplasmic	9.97	YES	YES	Virulence factors	YES
16	Q6GEE5	136	Stable	Cytoplasmic	9.97	YES	YES	Information and storage	YES
17	Q6GEK0	166	Stable	Cytoplasmic	9.97	YES	YES	Cellular process	NO
18	Q6GER8	368	Stable	Cytoplasmic	9.97	YES	YES	Metabolism Molecule	NO
19	Q6GEX6	146	Stable	Cytoplasmic	9.97	YES	YES	Metabolism Molecule	YES
20	Q6GFX2	329	Stable	Cytoplasmic	9.97	YES	YES	Metabolism Molecule	YES
21	Q6GGT4	219	Stable	Cytoplasmic	9.97	YES	YES	Cellular process	NO
22	A0A7U7ICS4	487	Stable	Cytoplasmic	9.97	YES	YES	Metabolism Molecule	NO
23	Q6GJH2	450	Stable	Cytoplasmic	9.97	YES	YES	Cellular process	YES
24	A0A7U7EVT9	452	Stable	Cytoplasmic	9.97	YES	YES	Virulence factors	NO
25	Q6GF03	382	Stable	Cytoplasmic	9.89	YES	YES	Metabolism Molecule	NO
26	A0A7U7EUV9	361	Stable	Cytoplasmic	9.89	YES	YES	Virulence factors	NO
27	A0A7U7EUI7	431	Stable	Cytoplasmic	9.68	YES	YES	Virulence factors	YES
28	A0A7U7IDV7	236	Stable	Cytoplasmic	9.67	YES	YES	Cellular process	NO
29	Q6GH12	240	Stable	Cytoplasmic	9.67	YES	YES	Cellular process	NO
30	Q6GIA4	313	Stable	Cytoplasmic	9.67	YES	YES	Metabolism Molecule	NO
31	A0A7U7ETI6	381	Stable	Cytoplasmic	9.67	YES	YES	Cellular process	NO
32	A0A7U7EVD2	260	Stable	Cytoplasmic	9.67	YES	YES	Metabolism Molecule	NO
33	Q6GEW9	179	Stable	Cytoplasmic	9.67	YES	YES	Cellular process	NO
34	Q6GF73	156	Stable	Cytoplasmic	9.67	YES	YES	Cellular process	NO
35	Q6GGH4	129	Stable	Cytoplasmic	9.67	YES	YES	Cellular process	NO
36	Q6GGJ5	271	Stable	Cytoplasmic	9.67	YES	YES	Virulence factors	NO
37	A0A7U7IDX4	221	Stable	Cytoplasmic	9.67	YES	YES	Cellular process	YES

#### 3.1.4 Heme response regulator R

Heme response regulator R (HssR) was considered an essential protein that manages heme levels in MRSA survival, virulence, and flexibility during the infection. HssR acts as a response regulator that detects the amount of heme inside the bacterial cell (Leasure et al., 2023). The physicochemical properties (Table 3) of HssR (UniProt ID: Q6GE73) comprised 224 amino acids, a molecular weight of 25,929.87 Da, and an isoelectric point of 5.87. The instability index was calculated to be 33.81, which classified this protein as stable. The Grand average of hydropathicity (GRAVY) was found to be −0.307, indicating that the protein was determined to be hydrophilic and soluble. The most abundant amino acid residue found in the HssR was leucine (10.7%), while the least abundant amino acid was tryptophan (0.4%). Further, the protein contains 32 negatively charged residues (aspartic acid + glutamic acid) and 29 positively charged residues (arginine + lysine). The atomic composition of this protein was found to be 3,672, with the molecular formula of C_1150_H_1852_N_316_O_344_S_10_.

**TABLE 3:** Physicochemical properties of the HssR.

S. No	Descriptions	Values
1	Number of amino acids	224
2	Molecular weight	25,929.87
3	Instability index	33.81
4	Aliphatic index	99.2
5	Theoretical pI	5.87
6	Formula	C_1150_H_1852_N_316_O_344_S_10_
7	Grand average of hydropathicity (GRAVY)	−0.307
8	Total number of atoms	3,672
9	Total number of negatively charged residues (Asp + Glu)	32
10	Total number of positively charged residues (Arg + Lys)	29

Further, the metabolic pathway of HssR was analyzed using the KEGG database. The various pathways and their corresponding KEGG IDs regulated by this protein were the bacterial secretion system (sau03070) and the two-component system (sau02020). From the analysis it revealed that these pathways were present only in the bacteria and not in humans, which indicated that this novel protein could n’t interfere with *H. sapiens* metabolic pathways. Further, protein-protein interaction (PPI) was investigated using the string database. It enables researchers to easily identify the network, biological processes, and nearby interacting proteins of HssR. The STRING database results are shown in Figure 2B. By analyzing the string database results, the HssR protein mainly acts as a hub protein that interacts with numerous MRSA-regulating proteins. So, targeting the HssR protein eventually disrupts the function of other interacting proteins, as these proteins interact with each other. The highly interacting proteins predicted through the database were HssS, HrtA, HrtB, staphylococcal respiratory response protein (SrrB), sensor histidine kinase (SaeS), and conserved hypothetical proteins (arlS and graS). Overall, the network consists of 11 nodes, 25 numbers of edges with an average local clustering coefficient of 0.715, and 10 expected numbers of edges. Hence, the HssR was found as a potential MRSA therapeutic drug target. Inhibiting the activity of this particular protein eventually reduces heme toxicity from MRSA infections, thereby increasing rate of removal immune cells and antibiotics.

### 3.2 Phase II

#### 3.2.1 Homology modeling and structure validation

**FIGURE 3:**
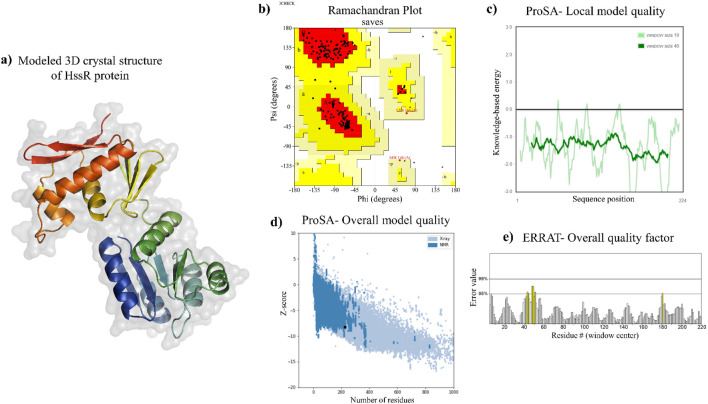
Modeled 3D structure of HssR and their validation. **(a)** predicted 3D structure of HssR using Swiss-model. **(b)** overall structure validation via Ramachandran plot [a = α-helix (right/left-handed); B = anti-parallel β-sheet; b = parallel β-sheet; p = proline]. The coloring and shading on the plot denote the permissible phi-psi backbone conformational regions, with the darkest portions (in red) indicating the most advantageous combinations of phi-psi values. **(c, d)** shows the local and overall model quality of HssR protein using ProSA webserver and **(e)** checking the overall quality factor using ERRAT sever.

The 3D crystal structure of the selected drug target of the MRSA was modeled using the Swiss Model Sever. It’s an automated protein homology modeling server that utilizes the protein FASTA sequence to model the protein of interest. The template was selected based on the quaternary structure quality estimate (GMQE) score, sequence similarity, sequence coverage, and oligo state of the template. By comparing various template structures in the AlphaFold protein structure database (AFDB), only the best model was determined as HssR protein by utilizing the Swiss Model webserver. The 3D structure of the predicted model is shown in Figure 3A, with the sequence identity of 80.98%, GMQE score of 0.91, sequence coverage of 1.0, and a biounit oligostate of monomer.

The modeled protein structure was verified using various web servers. The values are shown in Table 4. The Ramachandran plot predicted that the modeled protein had over 94.7% of residues in the most favored regions (Figure 3B), which indicated the predicted 3D structure to be a good model. Further, the Z-score of the model protein was determined to be −8.19 (Figures 3C, D), which also validated the modeled protein as more stable. The overall ERRAT quality factor was found to be 97.209% (Figure 3E), which suggested that the residues of the modeled protein provided a high-quality backbone structure. Subsequently, the modeled protein quality was also predicted by using the ProQ web server, which provides LGscore and MaxSub values of modeled structures. If the LGscore and MaxSub values were found to be greater than 4 and 0.5, then the modeled protein was declared as an extremely good model (Wallner and Elofsson, 2005). In this case, the HssR 3D structure had LGscore and MaxSub values of 10.456 and 0.5, respectively, an indication of the built protein to be an extremely good model. Hence, Ramachandran Plot analysis, ERRAT analysis, Z-score calculation, LGscore, and MaxSub value predictions suggested the modeled protein structure as a accurate and reliable model.

**TABLE 4:** 3D structure validations using various webservers.

Structure validation	Values	Webservers
Z-score	−8.19	ProSA
Overall quality factor	97.21%	ERRAT
LG score	10.456	ProQ
MaxSub value	0.5	
Residues in most favored regions	94.70%	ProCheck (Ramachandran Plot)
Residues in additional allowed regions	4.30%	
Residues in generously allowed regions	1%	
Residues in disallowed regions	0	

#### 3.2.2 Docking analysis

**FIGURE 4:**
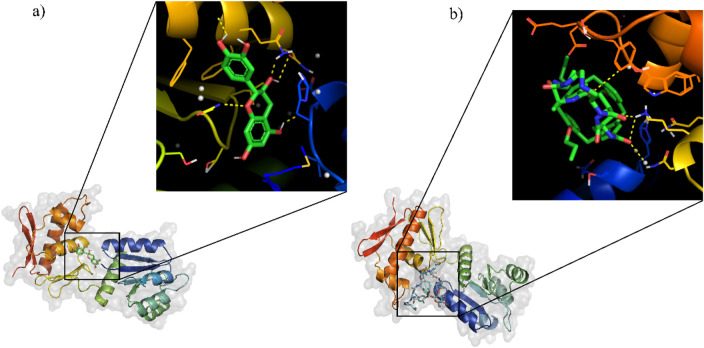
Molecular docking results of HssR protein with flavonoid compound and commercial drug. **(a)** Interaction of flavonoid compound (catechin) with the HssR protein and their binding sites. **(b)** interaction of reference drug (vancomycin) with the HssR protein and their binding site. The ligands are represented as green sticks while the yellow dashed line represent the hydrogen bond.

Many bioinformatics tools have been created and extensively utilized for the process of molecular docking, specifically in the drug development process. In this study, virtual screening were performed by utilizing ADV tools. Through the NuBBE database, a total of 173 flavonoid compounds were retrieved based on the selection criteria of drug-likeness rules, which include Lipinski’s rule of five, Veber’s rule, and Ghose’s rule (Table 5). These 173 compounds were docked against the HssR protein using virtual screening. From these results, the top ten compounds (Table 6) with low binding affinity complexes were selected to check the hydrogen bond interaction with HssR protein. Among these ten compounds, the Isolonchocarpin had low binding energy of −7.6 (kcal/mol^−1^), followed by 3′,4′-methylenedioxy-6,5-(2″,2″-dimethylpyran)-7-methoxyflavone, (+-)-7,4′-dihydroxy-3′-methoxyflavan and sulfuretin with binding energies of −7.2, −7.2, and −7.1 (kcal/mol^−1^) respectively. From the results of virtual screening, only one compound was determined to have the lowest binding affinity with more hydrogen bond interactions. The predicted compound was found to be catechin, which had a binding energy of −7.9 (kcal/mol^−1^) with 5 hydrogen bond interaction (GLU24, GLU136, GLU156, ASN152, and LYS177) hydrogen bond interactions. Also, the commercial antibiotic, vancomycin, had a binding energy of −5.9 (kcal/mol^−1^) with 4 hydrogen bond (SER19, LYS155, TRP179, and HIS193) interactions. Comparison of the docking results of both complexes (HssR-catechin and HssR-vancomycin) suggested that the compound catechin provided better binding affinity with the HssR protein than the commercial antibiotics. The 3D structures of HssR-catechin and HssR-vancomycin are shown in Figures 4A, B.

**TABLE 5:** Drug-likeness of compounds.

Properties of flavonoid compounds	Lipinski’s rule	Ghose rules	Veber’s rules
Molecular weight (Daltons)	≤500	160 to 480	
LogP	≤5	−0.4 to +5.6	
Hydrogen bond donors	≤5		
hydrogen bond acceptors	≤10		
Rotatable bonds			≤10
Polar Surface Area (Å^2^)			≤140
Molar Refractivity		40 to 130	

**TABLE 6:** Binding affinities of compounds computed through AutoDock and AutoDock Vina.

S. No	Compound ID	Compound name	Type of flavonoids	Binding energy AutoDock Vina (kcal/mol^−1^)	No. of hydrogen bond interaction
1	287	Catechin	Flavanol	−7.9	5
2	1,317	Isolonchocarpin	Flavanone	−7.6	1
3	1,589	3′,4′-methylenedioxy-6,5-(2″,2″-dimethylpyran)-7-methoxyflavone	Flavone	−7.2	2
4	2,224	(+-)-7,4′-dihydroxy-3′-methoxyflavan	Flavonoids	−7.2	1
5	567	Sulfuretin	Flavonoids	−7.1	2
6	1,320	7,8-(2″,2″-dimethylpyrano)-flavone	Flavone	−7	0
7	2035	5,2′-Dihydroxy-7-methoxy-6,8-dimethyl-4′,5′-methylenedioxyflavan	Flavonoids	−7	0
8	1,694	5,4′-dihydroxy-8,3′,5′-trimethoxy-6,7-(2″,2″-dimethylpyran)-flavone	Flavone	−6.9	2
9	1,305	3′,4′-methylenedioxy-5,7-dimethoxyflavone	Flavone	−6.8	0
10	1,777	2′-Hydroxy-7-methoxy-4′,5′-methylenedioxyflavan	Flavonoids	−6.8	0
11	Commercial Drug	Vancomycin		−5.9	1

#### 3.2.3 Molecular dynamic simulation

**FIGURE 5:**
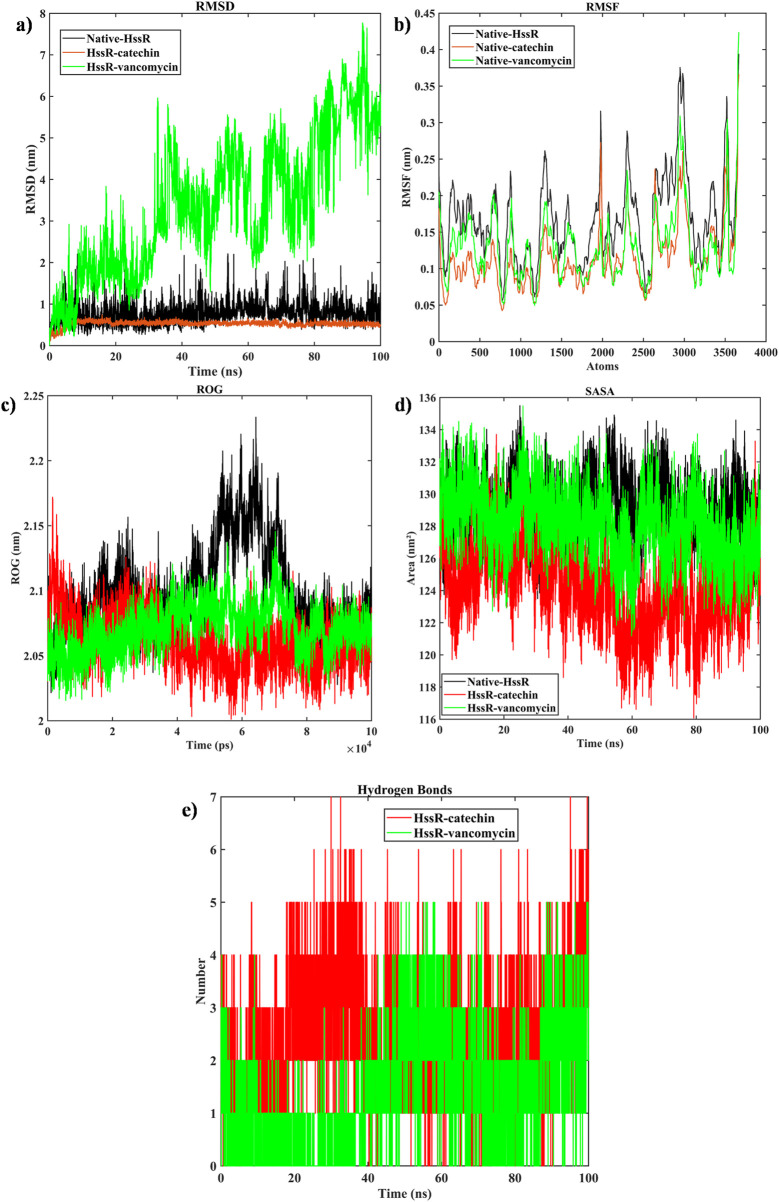
Evaluation of **(a)** RMSD, **(b)** RMSF, **(c)** ROG, **(d)** SASA, **(e)** Hydrogen bonds, (native protein (black), HssR-catechin complex (red) and HssR-vancomycin complex (green) during 100 ns simulation).

In order to evaluate the stability of the native proteins, HssR-catechin and HssR-vancomycin, the RMSD was estimated. Figure 5A shows the RMSD value of HssR and complexes. Initially, the RMSD value of the HssR-catechin complex was 0.3–0.5 nm; after 5 ns, the structure displayed stable fluctuation till the end of the 100 ns simulation. Similarly, the RMSD value of HssR-vancomycin was displayed in the range of 0.5–8 nm throughout 100 ns. The RMSD values suggested that HssR-catechin provided greater stability in contrast to HssR and HssR-vancomycin. Further, the RMSF analysis was carried out to understand whether the protein nature was flexible or rigid. Figure 5B shows the fluctuation of the backbone of the native proteins, HssR-catechin and HssR-vancomycin. The maximum RMSF value of native protein was found at 0.38 and 0.41 nm with atoms ranging from 2,950–3,020 and 3,510–3,580. Simultaneously, the RMSF of the HssR-catechin was determined at 0.28 and 0.37 nm within the atom range of 1990–2010 and 3,510–3,580, respectively, while the HssR-vancomycin was found at 0.31 and 0.42 nm in the atom range of 2,970–3,010 and 3,510–3,580, respectively. Therefore, the RMSF indicated that in HssR-catechin complex fluctuation were higher in the rigid regions than HssR and HssR-vancomycin. Following that, the structural compactness of the native protein and complex were calculated using the ROG analysis. Figure 5C illustrates the ROG value of native proteins as well as complexes. The ROG value of HssR-catechin was found in the range of 2.01–2.12 nm, the ROG value was observed in the range of 2.05–2.151 nm in HssR-vancomycin and the native HssR exhibited an ROG value in the range of 2.075–2.225 nm. Lesser the ROG value greater compactness of the protein (Tiwari et al., 2022). From this it confirmed that the catechin was more compact with HssR than other complexes or native protein. This demonstrated that the HssR-catechin was more stable in its folded state than that of HssR and HssR-vancomycin.

**FIGURE 6:**
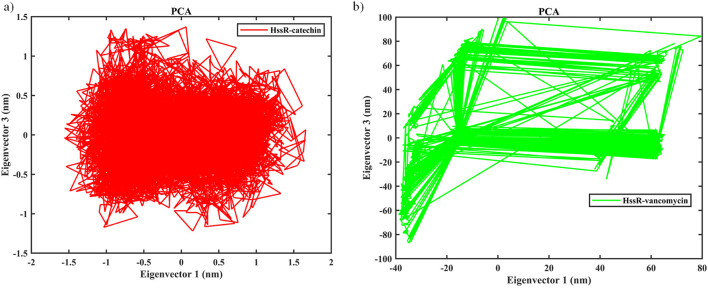
Evaluation of principal component analysis of complexes **(a)** PCA of HssR-catechin complex, and **(b)** PCA of HssR-vancomycin complex plots of native protein (black), HssR-catechin complex (red) and HssR-vancomycin complex (green) during 100 ns simulation. Binding free energy contribution of HssR-catechin and HssR-vancomycin from various interactions.

The contribution of ligand binding sites to the solvent behavior of the protein molecule was investigated using SASA. Figure 5D illustrates the SASA values of the native proteins and complexes. The SASA values of the native protein a had higher SASA value compared to HssR-catechin and HssR-Drug complex. The fluctuation for native protein was observed in the area of 160–165 nm^2^ during the 100 ns simulation. The SASA for the HssR-catechin complex was found in the lowest region of 120–130 nm^2^ compared to the HssR-vancomycin complex (130–137 nm^2^). By analyzing the SASA value, the HssR-catechin had the lowest surface area which indicates the complex is less exposed to the solvent. Subsequently, the hydrogen bond formations between the HssR protein and the ligands were measured for assessing the strength and stability of the complexes. Figure 5E displays the hydrogen bond formations. Throughout the 100 ns simulation, the chosen flavonoid complex exhibited 5 to 7 instances of hydrogen bond formations, while the commercial drug complex exhibited 4 to 5 instances of hydrogen bond formations. Hence, the flavonoids exhibited a greater propensity for hydrogen bond formation with HssR compared to commercial drugs. In addition, the principal component analysis (PCA) was analyzed to explore the prominent modes of motion in a trajectory. The eigenvalues and eigenvectors attained by diagonalizing the covariance matrix and the carbon-alpha motions of the two principal components (PC1 and PC2), which were further inspected by the essential dynamic method. Figures 6A, B show the 2D projection of trajectory plot generation in PCA. From the plot, the HssR-catechin complex occupied less space than HssR-vancomycin. It clearly indicated that the selected flavonoid compound catechin was more stable with HssR protein.

**FIGURE 7:**
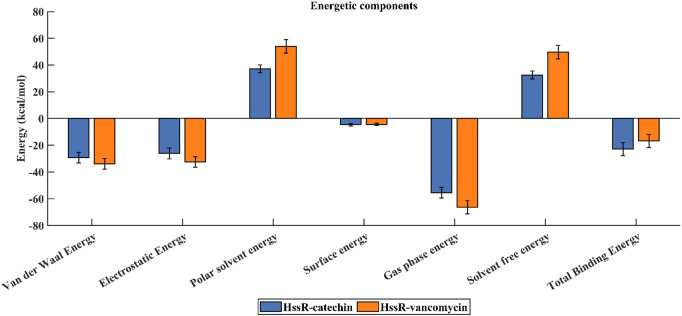
Binding free energy contribution of HssR-catechin (blue) and HssR-vancomycin (orange) from various interactions.

To assess the binding free energy of the simulated complexes, the last 20 ns of the trajectory was used (Valdés-Tresanco et al., 2021). The HssR-catechin and HssR-vancomycin complexes exhibited total binding free energies of −23.0 and −16.91 kcal/mol, respectively. The HssR-catechin complex was observed to have a stronger binding affinity between catechin and the HssR protein. Figures 7A, B illustrate the binding free energies of complexes, and Table 7 shows the energy contributions resulting from multiple interactions that led to the formation of both complexes.

**TABLE 7:** Computed binding free energies for complexes.

S. No	Energy component (kcal/mol^−1^)	Average		Standard deviation	
		HssR-catechin	HssR-vancomycin	HssR-catechin	HssR-vancomycin
1	Van der Waal energy	−29.26	−33.88	2.32	4.36
2	Electrostatic energy	−26.23	−32.58	4.51	8.05
3	Polar solvent energy	37.15	53.96	3.56	5.7
4	Surface energy	−4.66	−4.41	0.14	0.45
5	Gas phase energy	−55.49	−66.46	4.2	6.76
6	Solvent free energy	32.49	49.55	3.52	5.81
7	Total binding energy	−23	−16.91	2.53	4.6

The trajectory analysis of molecular dynamic simulation of catechin provided a stronger binding affinity and better stability with the HssR protein. Further, RMSD, RMSF, ROG, SASA, PCA, and H-bond results also depicts that the selected flavonoid complex (HssR-catechin) had more stability, flexibility, and high compactness compared to the commercial drug.

## 4 Discussion

The presence of various virulence proteins in MRSA causes severe healthcare-associated infections in every individual. Due to their multidrug-resistant properties, the infection couldn’t be easily controlled by the use of standard therapeutic drugs that were commercially available in the market. Overcoming this emergency by use of new antibacterial drugs that particularly target virulent proteins in MRSA becomes crucial. With the use of computational methods, prediction of the potential therapeutic targets and identification of therapeutic drugs will minimize the standardization initial in pharmaceutical research. In this study, the important virulence protein, namely, heme response regulator R (HssR), was predicted through the use of subtractive proteomic data analysis. The protein HssR was evaluated as an essential protein that highly responsible for regulating heme levels in MRSA.

After discovering HssR through the computational method, the 3D structure of this protein was built through homology modeling as their structure was not available in the RCSB repository. Further, the flavonoid compounds were retrieved to compute the binding affinity against HssR. The phytochemical compounds, especially flavonoids, had tremendous effects in combating the disease-causing proteins (Billowria et al., 2024). Due to their availability and ability to scavenging free radicals, reducing oxidative stress parameters, modulating inflammatory pathways and inhibiting the growth of microorganisms, flavonoids were chosen over other phytochemicals. Moreover, this study highlighted that flavonoid compound act as potential anti-MRSA agents that modulate several disease-causing metabolic pathways in MRSA infections. In this study, most of the flavonoid compounds had a binding energy above −6.0 kcal/mol, which indicated that the compounds had a greater inhibitory ability against HssR. Further, the compound catechin had the highest binding energy of −7.9 kcal/mol and also had 5 hydrogen bond interactions, whereas the commercial drug had the lowest binding affinity (−5.9 kcal/mol) and had only 4 hydrogen bond interactions. Furthermore, from others studies it was determined that the compound catechin had various properties, which include anti-microbial (Wang et al., 2022b), anti-tumor, anti-oxidant, anti-diabetic, anti-viral, anti-inflammatory (Baranwal et al., 2021; Gunti et al., 2019), and so on. In a recent study, the researchers used a catechin compound from a cashew nut shell to combat ATCC and clinical isolates of MRSA. The results revealed that the catechin greatly damaged the bacterial cell wall and increased reactive oxygen species, which indicated that the compound potentially acted as an anti-MRSA agent (Sinsinwar and Vadivel, 2020). With these enormous properties reported by researchers, the catechin utilized in this study also proved that it had the strongest ability to modulate the activity of a specific MRSA therapeutic target, namely, HssR, which is known to regulate the heme levels in MRSA infections. With these findings, further *in vitro* and *in vivo* studies will be evaluated to assess the inhibition ability of catechin against the HssR virulent protein. Moreover, this investigation could be utilized for inhibiting the proliferation and activation of *Staphylococcus* microorganisms that were considered harmful to humans.

Further, the results of AutoDock Vina were determined that the flavonoid compounds had a strongest binding affinity than vancomycin. In another research, a list of polyhydroxylated flavonoids were used against MRSA protein, namely, penicillin-binding protein 2a, and determined the binding energies through AutoDock Vina and the neural networking method. All these methods provided a binding affinity of above −7.0 kcal/mol, which suggested that the flavonoid compounds used in this study were a great choice for inhibiting the virulence effects of MRSA (Verma et al., 2022). In our previous study, traditional medicine compounds, with a wide variety of phenolic compounds, were docked against staphylococcal scaled skin syndrome-causing protein, namely, exfoliative toxin B, and found that the docking scores were greater than −7.6 kcal/mol (Kumar Subramani and Venugopal, 2024). In comparison particular types of phenolic compounds, specifically flavonoids, provided better binding interactions with MRSA disease-causing proteins in this study.

Furthermore, the analysis of molecule movement through the use of molecular dynamic simulations also suggested that the catechin compound provided more stable compatibility with HssR protein than vancomycin during the 100 ns simulations. In addition to that, RMSD, RMSF, ROG, PCA, SASA, hydrogen bond interactions and binding free energy calculation also validated that the catechin had a stronger correlation with HssR than vancomycin. In conclusion, the binding-free calculation between the two complexes, which includes catechin-HssR and vancomycin-HssR, also confirmed that the flavonoid compounds could act as a potential anti-MRSA agent against HssR.

## 5 Conclusion

The novel virulence factor, namely, heme response regulator R (HssR), that causes MRSA infection in humans was predicted through subtractive proteomic data analysis from a vast set of proteins. Subsequently, the flavonoid compound catechin demonstrated higher binding activity against HssR than vancomycin. Moreover, the RMSD, RMSF, ROG, hydrogen bond prediction, SASA, PCA, and binding free energy calculations through molecular dynamic simulations corroborated the results of this study. Overall, the outcome of this *in-silco* study confirms that catechin had the potential to be exploited as an alternative anti-MRSA agent that combats microbial infections caused by *Staphylococcus*.

## Data Availability

The datasets presented in this study can be found in online repositories. The names of the repository/repositories and accession number(s) can be found in the article/Supplementary Material.

## Abbreviations

MRSA, Methicillin Resistant *Staphylococcus aureus*; HssR, Heme response regulator R; PBPs, Penicillin-binding proteins; ADV, AutoDock Vina; MDS, Molecular Dynamic Simulation; RMSD, Root Mean Square Deviation; RMSF, Root Mean Square Fluctuation; ROG, Radius of Gyration; H-bond, Hydrogen Bond; SASA, Solvent Accessible Surface Area; PCA, Principal Component Analysis; MMGBSA, Molecular mechanics with generalized Born and surface area solvation.
